# Investigation of blood leptin and adropin levels in patients with multiple sclerosis: A CONSORT-clinical study

**DOI:** 10.1097/MD.0000000000027247

**Published:** 2021-09-17

**Authors:** Ufuk Cinkir, Levent Sinan Bir, Senay Topsakal, Esin Avci Cicek, Selma Tekin

**Affiliations:** a Basaksehir Cam and Sakura City Hospital, Istanbul, Turkey; b Pamukkale University Faculty of Medicine, Denizli, Turkey.

**Keywords:** adipokine, biomarker, inflammation, mammalian target of rapamycin, neurodegeneration

## Abstract

**Background:**

The effects of adipokines have been investigated in multiple sclerosis (MS) in the literature. Results are uncertain, and subgroups like adropin have not been previously studied. We primarily aimed to determine leptin and adropin levels in MS and their potential use as a biomarker.

**Methods:**

This study was an experimental research. While 44 MS patients diagnosed according to McDonald criteria were included in the patient group, 40 people without MS diagnosis and risk factors took part in the control group. Demographic data, height, weight, body mass index, blood glucose, thyroid-stimulating hormone, alanine transaminase, aspartate transaminase, creatinine, low-density lipoprotein, leptin, adropin levels, presence of hypertension, diabetes mellitus, coronary artery disease were recorded. Expanded disability status scale and disease duration were also evaluated in the patient group. Our data were presented as mean ± standard deviations.

**Results:**

The mean blood leptin value of the patient group (6.12 ± 5.34 ng/mL) was significantly lower than the value of the control group (13.02 ± 8.25 ng/mL) (*P* < .001). The patient group had a mean adropin level of 504.12 ± 311.17 ng/mL, which was significantly lower than that of the control group (747.0 ± 309.42 ng/mL) (*P* < .001). Statistically insignificant differences were found between their body mass index, glucose, alanine transaminase, aspartate transaminase, thyroid-stimulating hormone, low-density lipoprotein levels (*P* > .001).

**Conclusion:**

This is the first study that has evaluated adropin levels in patients with MS. The relationship between MS and leptin levels is still unclear. Therefore, our study might be helpful to elucidate MS pathogenesis and provide supportive criteria for diagnosis.

## 1 Introduction

MS is a chronic disease with neurodegeneration and inflammation that can present various signs and symptoms. It generally affects younger adults and is characterized by inflammation, demyelination, and axonal degeneration of the central nervous system (CNS).^[1,2]^ MS may cause severe progressive disability.^[1,2]^ MS is more common in Caucasians, women, temperate, and high-income populations.^[1,2]^ In addition, it is thought to affect approximately 2 million people worldwide.^[1,2]^ A variety of lesions are observed in both white and grey matter of MS patients, which may cause several symptoms.^[3,4]^ The most common are loss of sensation, numbness, loss of motor strength and vision, dizziness, imbalance, diplopia, and bladder problems.^[3,4]^ Cognitive dysfunction and spasticity are considered as an indication of disease progression.^[3,4]^ That is, a wide variety of signs and symptoms may be seen due to the lesions.

Adipokines are among the hormones whose effects have been investigated in MS for several years.^[5–8]^ Leptin is an adipokine secreted from white adipose tissue and virtually affects the neuroendocrine system and immunomodulation.^[5]^ Some previous studies focused on the relationship between leptin levels and MS, yet those earlier studies are still inconsistent.^[6–8]^ Xie et al^[7]^ found that higher serum leptin levels were found in MS patients compared with the control group. However, in 2018, Kvistad et al^[8]^ demonstrated leptin and adiponectin were not useful as biomarkers of MS activity.

Adropin is a peptide hormone produced in the liver and brain^[9,10]^ involved in energy homeostasis, glucose and fatty acid metabolism,^[11,12]^ found at high concentrations in the brain and involved in developing the cerebellum.^[13]^ We decided to investigate the level of adropin in MS, a neurodegenerative disease, due to its potential neuroprotective effects in the central nervous system. There is no study on adropin levels in MS so far.

We aim to determine leptin and adropin levels in MS patients, any potential relationship between leptin and adropin levels with the disease. Unlike previous studies, in this present original study, we studied whether there is any possible relationship between adropin levels and MS for the first time. To the best of our knowledge, this study may be a pioneer in the field.

## 2 Methods

### 2.1 Ethical review

The committee approved the ethical issues of the present study of Pamukkale University non-invasive clinical research ethics.

### 2.2 Participants

Definite MS patients, diagnosed according to McDonald MS criteria, were included in the patient group who had been evaluated in the department of neurology in the medical faculty (n = 44) of Pamukkale University, Denizli, Turkey. All patients were in remission but not in an attack period. The time elapsed since the last attack was unknown. People without MS diagnosis and risk factors (MS family history, coexisting certain autoimmune diseases, and infections such as systemic lupus erythematosus, Sjogren syndrome, Epstein-Barr virus infection, Lyme disease) were included in the control group. During the participants’ selection, all members were informed to participate in the study, and informed consent was obtained from each participant.

In each group, demographic data, height, weight, body mass index, blood glucose level, thyroid-stimulating hormone, alanine transaminase, aspartate transaminase, creatinine, low-density lipoprotein, leptin, adropin levels were measured, and present hypertension and diabetes mellitus, coronary artery disease was also recorded. Expanded disability status scale (EDSS) and disease duration were also determined in the patient group.

### 2.3 Blood collection

After obtaining informed consents, 5 mL of blood taken from the median cubital vein was transferred into yellow capped serum tubes containing separating gel. After blood was centrifuged (1500 × *g* for 10 minutes at +40 °C), serum was separated and stored in Eppendorf tubes at –80 °C prior to analyses.

### 2.4 Quantification of leptin and adropin

Adropin and leptin levels were determined by Y.L. Biont (Shangai Y.L. Biotech Co. Ltd, China) kits using enzyme-linked immunosorbent assay from the sera brought to room temperature on the day of analyses. Absorbance readings were carried out at a wavelength of 450 nm with a BioTek brand Enzyme-linked immunosorbent assay reader, and concentrations were calculated using the Gen 5 program. Within-run coefficient of variation levels for adropin and leptin were <8%, and inter-trial coefficient of variation <10%. Adropin reading range was between 5 and 1000 ng/L, while the kit's sensitivity was 2.49 ng/L. The reading range of leptin was between 20 and 8000 pg/L, while the kit's sensitivity was 10.83 pg/L.

### 2.5 Study design

Our study was experimental and cross-sectional clinical research.

### 2.6 Statistical analysis

Data were analyzed by the IBM SPSS 25 program (Armonk, New York, USA). As a result of the power analysis, it was calculated that if there were at least 29 people in the patient and control group, its validity would be achieved with a power of 80% with 95% confidence, and an independent sample *t* test was applied where parametric test assumptions were met. The Mann–Whitney *U* test was used for variables where parametric test assumptions were not provided. *P* ≤ .01 was considered significant. Our data were presented as mean ± standard deviations.

## 3 Results

The mean age distribution of both groups was 39.71 ± 11.10. Of the all participants, there were 4 (4.8%) hypertensive and 80 (95.2%) non-hypertensive; 9 (10.7%) diabetic and 75 (89.3%) non-diabetic; 2 (2.4%) with coronary artery disease and 82 (97.6%) without coronary artery disease; 16 (19%) with hyperlipidemia and 68 (81%) participants without hyperlipidemia (Table 1).

**Table 1:** The demographic and physical data of all the participants.

Variable	Mean ± standard deviation	Median (minimum–maximum)
Age, yr	39.71±11.10	40 (18–75)
Length, cm	165.26 ± 9.06	164 (150–191)
Weight, kg	71.15 ± 13.83	70 (40–110)
Body mass index	26.04 ± 4.76	25.39 (15.6–38.2)

		n (%)
Gender	Women	61 (72.6%)
	Men	23 (27.4%)
Hypertension	Yes	4 (4.8%)
	No	80 (95.2%)
Diabetes mellitus	Yes	9 (10.7%)
	No	75 (89.3%)
Coronary artery disease	Yes	2 (2.4%)
	No	82 (97.6%)
High LDL level	Yes	16 (19%)
	No	68 (81%)

LDL = low density lipoprotein.

Of the patient group in our study, 34 (77.3%) were women, 10 (22.7%) were men, and the female/male ratio was 3.4, which was compatible with the literature (Supplemental Digital Content 1).

The mean EDSS of the patient group was 2.97 ± 2.17, and the mean disease duration was 9.58 ± 7.20; the mean number of attacks was 7.7 ± 8.4. There were 1 clinically isolated syndrome (2.3%), 37 relapsing-remitting MS (RRMS) (84.1%), 6 secondary progressive MS (SPMS) (13.6%) cases (Table 2).

**Table 2:** The information about the disease in the patient group.

Variable	Mean ± standard deviation	Median (minimum–maximum)
EDSS	2.97 ± 2.17	2 (0–9)
Disease duration, y	9.58 ± 7.20	7 (3–42)
Number of attacks	7.7 ± 8.47	4.5 (1–30)

		n (%)
MS form	RRMS	37 (84.1%)
	SPMS	6 (13.6%)
	CIS	1 (2.3%)

CIS = clinical isolated syndrome, EDSS = expanded disability status scale, MS = multiple sclerosis, RRMS = relapsing-remitting multiple sclerosis, SPMS = secondary progressive multiple sclerosis.

The mean blood leptin value of the patient group was 6.12 ± 5.34 ng/mL. In comparison, it was 13.02 ± 8.25 ng/mL for the control group, and the difference was statistically significant (*P* < .001) (Table 3, Supplemental Digital Content 2). The mean adropin levels of the patient and the control groups were 504.12 ± 311.17 and 747.0 ± 309.42 ng/mL, respectively. And, the difference was statistically significant (*P* < .001) (Table 3, Supplemental Digital Content 3). Leptin and adropin values did not show any statistically significant difference between RRMS and SPMS groups (*P* < .001). The relationship between EDSS and leptin and adropin in the RRMS and SPMS groups was statistically insignificant (*P* > .001).

**Table 3:** The laboratory and physical data of the patient and control group.

Variable	Patient group (n = 44)	Control group (n = 40)	*P*
Age	40.75 ± 10.76	38.58 ± 11.49	.375
Height	164.14 ± 9.0	166.5 ± 9.06	.235
Weight	68.95 ± 14.62	73.58 ± 12.65	.124
BMI	25.63 ± 5.23	26.51 ± 4.20	.404
Glucose	98.30 ± 20.90	102.33 ± 26.04	.439
TSH	2.05 ± 1.75	1.76 ± 0.81	.330
ALT	24.25 ± 22.66	16.88 ± 10.77	.058
AST	20.61 ± 12.94	15.68 ± 4.84	.022
Creatinine	0.69 ± 0.17	0.78 ± 0.15	.012
LDL	110.87 ± 31.51 (n = 38)	119.68 ± 38.33 (19)	.393
Leptin	6.12 ± 5.34	13.02 ± 8.25	.0001
Adropin	504.12 ± 311.17	747.0 ± 309.42	.0001

ALT = alanine transaminase, AST = aspartate transaminase, BMI = body mass index, LDL = low density lipoprotein, TSH = thyroid-stimulating hormone.

## 4 Discussion

This study is the first to evaluate adropin levels in MS patients to the best of our knowledge. We investigated whether adropin and leptin had a potential role in MS and whether they could be used as biomarkers by looking at blood adropin and leptin levels in patients with MS and healthy control groups.

MS is a chronic inflammatory and degenerative CNS disease that mainly affects young people. It is more common in White, women, temperate climates, and high-income societies.^[1]^ It is one of the leading causes of disability in young and middle-aged people in developed countries.^[1]^ Among the prevalence studies, it was reported that the MS prevalence was 101.4 /100,000 in the study in the Maltepe district of Istanbul and 33.9 /100,000 in the study in Edirne.^[14,15]^

The mean age in Turkey has been reported as 41.8 ± 12.0 years, and in a study conducted in the Thrace region, it was found as 40.7 ± 10.6 years.^[14,16]^ Similarly, the patients’ mean age in our study was 40.75 ± 10.76 years. MS is more common among women. Worldwide, women with MS are about twice as high as men.^[4]^ Of the patient group in our study, 77.3% were female, 22.7% male and the female/male ratio (3.4) was compatible with the literature.

In a study conducted on the prevalence of DM, hyperlipidemia, and hypertension in MS patients, the rate of hyperlipidemia in MS patients was not different from the average population.^[17]^ Similarly, the patient group's rate of hyperlipidemia was not statistically significant in our study compared with the control group.

Leptin is primarily secreted from white adipose tissue. It acts on the brain and peripheral tissues. It plays a role in food intake, energy expenditure, metabolism, neuroendocrine axis, and immune function. It is involved in energy homeostasis in the brain. It is synthesized in the placenta, ovaries, skeletal muscles, pituitary gland, lymphoid tissue, and white adipose tissue.^[18]^ Levels of leptin in the circulation increase in proportion to the amount of fat in the body and indicate long-term energy stores. It decreases in hunger and is affected by changes in calorie intake.^[19]^ While it increases in overnutrition and obesity, it falls in hunger and low-calorie intake.^[19]^ Insulin, glucocorticoids increase leptin levels while catecholamines decrease.^[20]^

Leptin receptor long isoform is involved in energy homeostasis of the brain, hedonic regulation of nutrition, neuroendocrine function, memory, and learning.^[21]^ A pathway generated by leptin is the mammalian target of rapamycin (mTOR). mTOR is the downstream target of phosphatidylinositol 3-kinase (PI3K)/Protein kinase B (Akt). Inhibition of mTOR reduces the anorexigenic effect of leptin.^[39]^ So leptin activates mTOR. Leptin was not found helpful as an MS activity biomarker in one study^[8]^ but, in a meta-analysis, higher serum leptin levels were found in MS patients than the control group,^[7]^ suggesting leptin as a biomarker. The role of leptin could not be demonstrated in a study conducted with naive patients with clinically isolated syndrome and RRMS.^[22]^ Another study showed baseline serum leptin levels in the control and MS groups. In the first year of interferon beta-1a treatment, leptin decreased in 11 patients without relapse 2 months after starting treatment. In 13 patients, leptin increased before the first clinical exacerbation. So, leptin might be useful as a biomarker.^[23]^ The relationship between MS and leptin is unclear in the literature. Unlike other publications, in our study, the patient group had a leptin level significantly lower than the control group (*P* < .001), most probably because leptin activates the mTOR pathway. Thus, it increases angiogenesis, neuronal regeneration, synaptic plasticity. In other words, it contributes positively to the functions of neurons.^[24,25]^ The decrease in leptin may impair angiogenesis, regeneration, and the formation of synaptic plasticity. So, this could contribute to neuronal damage. The scarcity of leptin may lead to MS disease with inflammation and neurodegeneration and perhaps even pave the way.

Adropin is a peptide hormone produced in the liver and brain.^[9,10]^ It is encoded by the Enho gene. The Enho transcript expression has been reported in the liver and brain.^[9,26]^ Recent data have shown that adropin is a protein involved in energy homeostasis, glucose, and fatty acid metabolism.^[11,27]^ Adropin has glucose and insulin sensitivity regulatory functions^[9,10]^ and endothelial protective potential.^[10]^ It is found in the brain at high concentrations and has a role in developing the cerebellum.^[13]^ Decreased physical activity, abnormal coordination and motor activity, and impaired formation of cerebellum synapses were observed in mice whose adropin gene was removed.^[28]^ Adropin supports neurogenesis.^[28,29]^ Vascular endothelial growth factor receptor 2 is released explicitly from the endothelium, regulating endothelial function and angiogenesis. Adropin upregulates this receptor and activates the PI3K/Akt and the extracellular-signal-regulated kinase (ERK)1/2 pathways, accelerating endothelial nitric oxide synthase. In experimental studies, inhibition of the extracellular-signal-regulated kinase 1/2 pathway is associated with oxidative stress and endothelial dysfunction in the brain.^[10,30]^ Hypoxia accelerates hypoxia-inducible factor 1a and vascular endothelial growth factor gene expression.^[31]^

Neurodegenerative diseases such as Alzheimer, Parkinson, and Huntington disease are associated with the Akt signal defect.^[16,32–34]^ Hence, PI3K/Akt activation by adropin may have therapeutic potential in neurodegenerative disorders.^[33,35]^ Adropin activates Akt by phosphorylation induction.^[10]^ Phosphorylated-Akt ensures cell cycle, proliferation, differentiation, and survival.^[36,37]^ This path also triggers the mTOR pathway. mTOR is vital in angiogenesis, neuronal regeneration, synaptic plasticity, inflammatory responses, apoptosis.^[24,25,38]^ Again, like leptin, low adropin levels may impair angiogenesis and regeneration and disrupt synaptic plasticity. In our study, the patient group had a level of adropin significantly lower than the control group (*P* < .001), which may lead us to the fact that adropin deficiency may promote inflammation and neurodegeneration in the CNS and/or contribute to the formation of MS. There has not been any study investigating the relationship between MS and adropin in the literature. Our results indicated that based on the significant difference in the adropin levels between the 2 groups, adropin levels might be used as a biomarker in MS patients. Besides, considering its role in energy metabolism, the effects of increased adropin levels on the potential treatments for neurodegenerative diseases like MS should be determined in future studies.

On the other hand, we had some limitations in our study. Firstly, there was no primary progressive MS in our patient population. Secondly, our population's count was quite limited (84 participants). The last limitation was that we did not evaluate patients and biochemical parameters according to disease subgroups. Larger patient groups, including primary progressive MS and divided subgroups, should be studied further.

In conclusion, the relationship between MS and leptin level is not clear in the literature. This study is the first on the potential relationship between MS and blood adropin levels to the best of our knowledge. The role of blood leptin and adropin levels should be taken into consideration in patients with MS. Their levels may be used as a predictive value for MS in the future and can provide valuable information about the course of this disease.

## Author contributions

**Conceptualization:** Ufuk Cinkir.

**Data curation:** Ufuk Cinkir.

**Formal analysis:** Ufuk Cinkir, Senay Topsakal, Esin Avci Cicek.

**Funding acquisition:** Ufuk Cinkir, Senay Topsakal.

**Investigation:** Ufuk Cinkir, Levent Sinan Bir, Senay Topsakal, Esin Avci Cicek.

**Methodology:** Ufuk Cinkir, Levent Sinan Bir, Senay Topsakal, Esin Avci Cicek.

**Project administration:** Ufuk Cinkir.

**Resources:** Ufuk Cinkir, Esin Avci Cicek.

**Supervision:** Ufuk Cinkir, Selma Tekin.

**Validation:** Ufuk Cinkir.

**Visualization:** Ufuk Cinkir.

**Writing – original draft:** Ufuk Cinkir, Senay Topsakal, Esin Avci Cicek, Selma Tekin.

**Writing – review & editing:** Ufuk Cinkir, Levent Sinan Bir, Esin Avci Cicek, Selma Tekin.

## Supplementary Material

Supplemental Digital Content

## Supplementary Material

Supplemental Digital Content

## Supplementary Material

Supplemental Digital Content
