# Supplementary figures and images for: Investigation of blood leptin and adropin levels in patients with multiple sclerosis: A CONSORT-clinical study
Source: Medicine (Baltimore). 2021 Sep 17;100(37):e27247. doi: 10.1097/MD.0000000000027247 (PMC8448068; doi:10.1097/MD.0000000000027247)

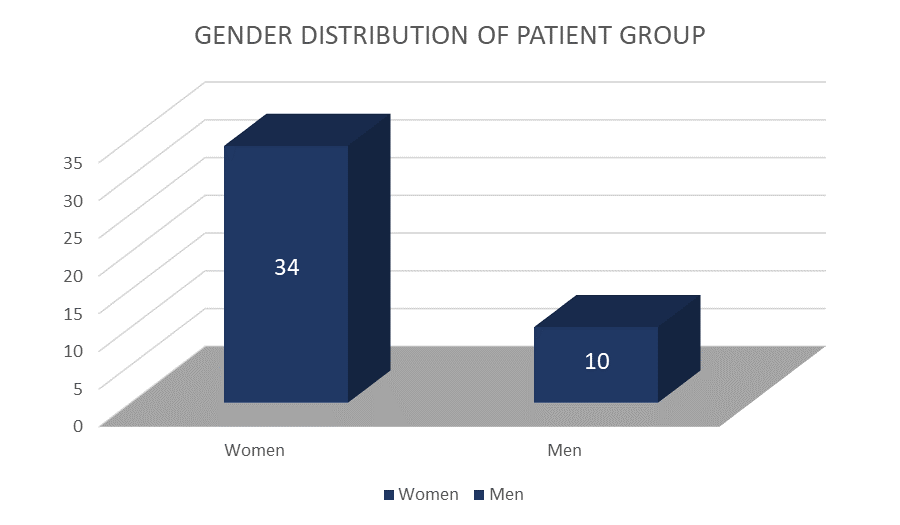

Supplement: Supplemental Digital Content [file medi-100-e27247-s001.docx]

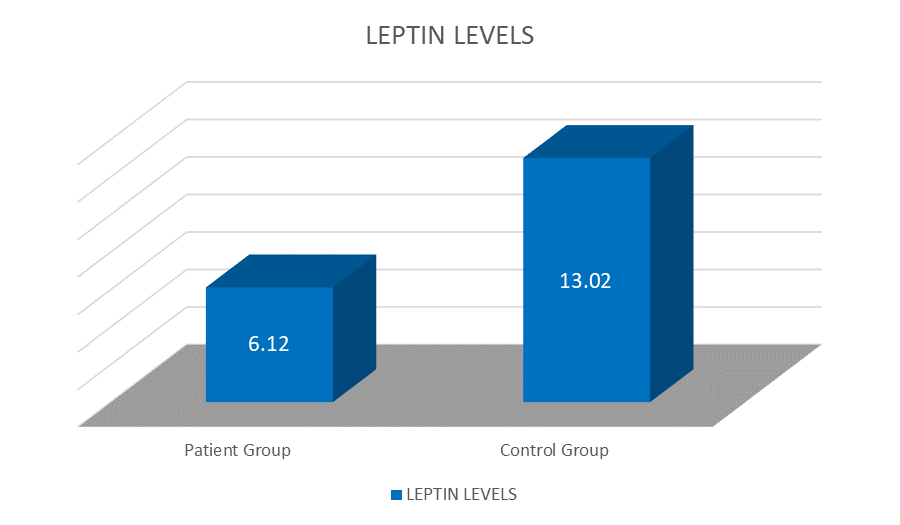

Supplement: Supplemental Digital Content [file medi-100-e27247-s002.docx]

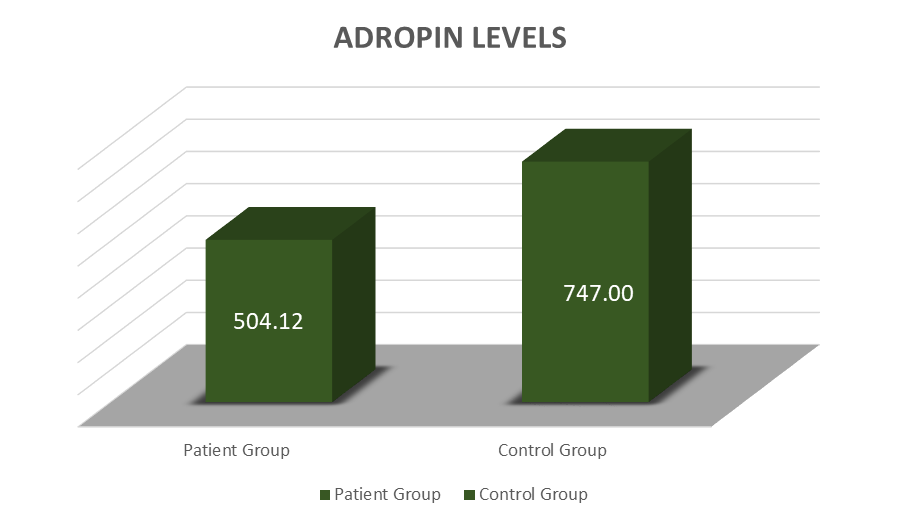

Supplement: Supplemental Digital Content [file medi-100-e27247-s003.docx]
